# An update on the potential mechanism of gallic acid as an antibacterial and anticancer agent

**DOI:** 10.1002/fsn3.3615

**Published:** 2023-08-31

**Authors:** Saeedeh Keyvani‐Ghamsari, Maryam Rahimi, Khatereh Khorsandi

**Affiliations:** ^1^ Clinical Cares and Health Promotion Research Center, Karaj Branch Islamic Azad University Karaj Iran; ^2^ Department of Photodynamic, Medical Laser Research Center Yara Institute, ACECR Tehran Iran

**Keywords:** antibacterial, anticancer, gallic acid, health benefits, polyphenol

## Abstract

Drug resistance to antibacterial and anticancer drugs is one of the most important global problems in the treatment field that is constantly expanding and hinders the recovery and survival of patients. Therefore, it is necessary to identify compounds that have antibacterial and anticancer properties or increase the effectiveness of existing drugs. One of these approaches is using natural compounds that have few side effects and are effective. Gallic acid (GA) has been identified as one of the most important plant polyphenols that health‐promoting effects in various aspects such as bacterial and viral infections, cancer, inflammatory, neuropsychological, gastrointestinal, and metabolic disease. Various studies have shown that GA inhibits bacterial growth by altering membrane structure, and bacterial metabolism, and inhibits biofilm formation. Also, GA inhibits cancer cell growth by targeting different signaling pathways in apoptosis, increasing reactive oxygen species (ROS) production, targeting the cell cycle, and inhibiting oncogenes and matrix metalloproteinases (MMPs) expression. Due to the powerful function of GA against bacteria and cancer cells. In this review, we describe the latest findings in the field of the sources and chemical properties of GA, its pharmacological properties and bioavailability, the antibacterial and anticancer activities of GA, and its derivatives alone, in combination with other drugs and in the form of nanoformulation. This review can be a comprehensive perspective for scientists to use medicinal compounds containing GA in future research and expand its clinical applications.

## Graphical abstract

Gallic acid (GA) has been identified as one of the most important plant polyphenols that have health‐promoting effects in various aspects. Various studies have shown that GA inhibits bacterial growth by altering membrane structure, and bacterial metabolism, and inhibits biofilm formation and growth. GA inhibits cancer cell growth by targeting different signaling pathways in apoptosis, increasing reactive oxygen species (ROS) production, targeting the cell cycle, and inhibiting oncogenes and matrix metalloproteinases (MMPs) expression.

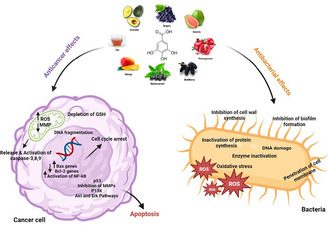

## 1 INTRODUCTION

Nowadays, the growing resistance to antibiotic and anticancer drugs and treatment failure leads to increased economic and treatment costs besides long‐term hospitalization, which requires finding new approaches. Given the importance of antibiotic resistance, the World Health Organization (WHO) predicts that if this problem is not addressed, deaths from resistant bacterial infections will be the leading cause of death in the world by 2050 (Manso et al., 2021) Increased resistance of pathogenic microorganisms has often been attributed to inappropriate use of antibiotics and transmission of resistance within and between individuals. Given that the production of new antibiotics in the industry has not attracted the attention of investors and is not cost‐effective, new strategies are needed to prevent the emergence and spread of drug resistance, inhibit bacterial growth, and prolong the life of conventional antibiotics (Minarini et al., 2020; Prestinaci et al., 2015).

On the other hand, resistance to anticancer drugs has limited the effectiveness of existing treatments for cancer. Drug resistance can develop as inherent resistance of cancer cells to the drug or as acquired during treatment which the growth of resistant cells leads to treatment failure (Holohan et al., 2013). Therefore, overcoming resistance can increase the efficiency of treatment.

Recently, the use of natural compounds to control and overcome drug resistance in cancer cells as well as against bacteria has been considered by researchers (Álvarez‐Martínez et al., 2020; Keyvani‐Ghamsari et al., 2020). Among the natural compounds, phenolic compounds in plants are considered secondary plant metabolites and have one or more aromatic rings with hydroxyl groups. So far, about 8000 phenolic compounds are known, including tannins, flavonoids, ligands, coumarins, and xanthines. Many of them have anticancer and antibacterial effects (Curti et al., 2017; Khorsandi et al., 2020). Gallic acid (GA) is one of the most important natural polyphenols. Carl Wilhelm Scheele, a Swedish chemist, first isolated GA from plants in 1786, after which studies began on the function of GA and its derivatives (Yang, Zhang, et al., 2020). It is a natural secondary plant metabolite with extensive biological activities such as anti‐inflammatory, antibacterial, antifungal, antiulcerogenic, and anticancer (Fernandes & Salgado, 2016) (Figure 2). In addition, due to its ability to inhibit free radicals, it is a powerful antioxidant used as a preservative in food packaging ingredients, prepared and processed foods, beverages, drugs, and cosmetics to prevent the effects of peroxidation and fat breakdown (Yen et al., 2002).

GA and its derivatives alone and in combination with other drugs can prevent the growth of planktonic and biofilm of different types of bacteria by various mechanisms (Subramanian et al., 2016). Also, GA in cancer cells can induce apoptosis by affecting the expression of oncogenes, apoptotic proteins, antiapoptosis, matrix metalloproteinases (MMPs), ROS production, and targeting the cell cycle (Moghtaderi et al., 2018; Subramanian et al., 2016; Yang et al., 2022).

In this review, we describe the antibacterial and anticancer function of GA and its derivatives alone, in combination with other drugs, and in the nanoparticle formulation against various cancer cells and bacteria.

## 2 THE SOURCES AND CHEMICAL PROPERTIES OF GA AND ITS DERIVATIONS

GA (3, 4, 5‐trihydroxybenzoic acid) with the chemical formula of C_6_H_2_(OH)_3_COOH is an organic acid that is known as a powerful antioxidant in plants, vegetables, and fruits. It is one of the most abundant phenolic acids in herbs and foods (Moghtaderi et al., 2018). GA is present in the roots, skin, stems, leaves, flowers, and seeds of plants mainly in the form of ester derivatives combined with sugars, phenols, and polyols (Badhani et al., 2015). It is present in various components of medicinal plants such as *Phyllanthus amarus* (Euphorbiaceae), *Mentha spicata* (Lamiaceae), *Abutilon pictum* (Malvaceae), *Achillea millefolium* (Asteraceae), and *Momordica cabrae* (Cucurbitaceae) (Bai et al., 2021). The amount of GA extracted from plants is very different and ranges from 0.001 to 135.08 mg/g. The highest levels of it were found in *Phyllanthus A*., followed by *Momordica C*. and *Achillea S*. (Zhang, Liu, et al., 2020). It is also naturally present in dietary substances such as sundew, blackberry, bearberry, gallnut, vinegar, tea leaves, cloves, sumac, hot chocolate, and beverages such as red wine, and green tea (Subramanian et al., 2015).

It is a yellowish‐white crystalline compound with a molecular mass of 170.12 g/mol, a melting point of 210°C, pKa of 4.40, a density of 1.69 kg/L (20°C), and a solubility of 1.1% in water at 20°C and soluble in alcohol, ether, acetone, and glycerol (Choubey et al., 2015). GA is widely used in pharmacy, cosmetics, the food industry, and medical and chemical research (Brewer, 2011).

**FIGURE 1:**
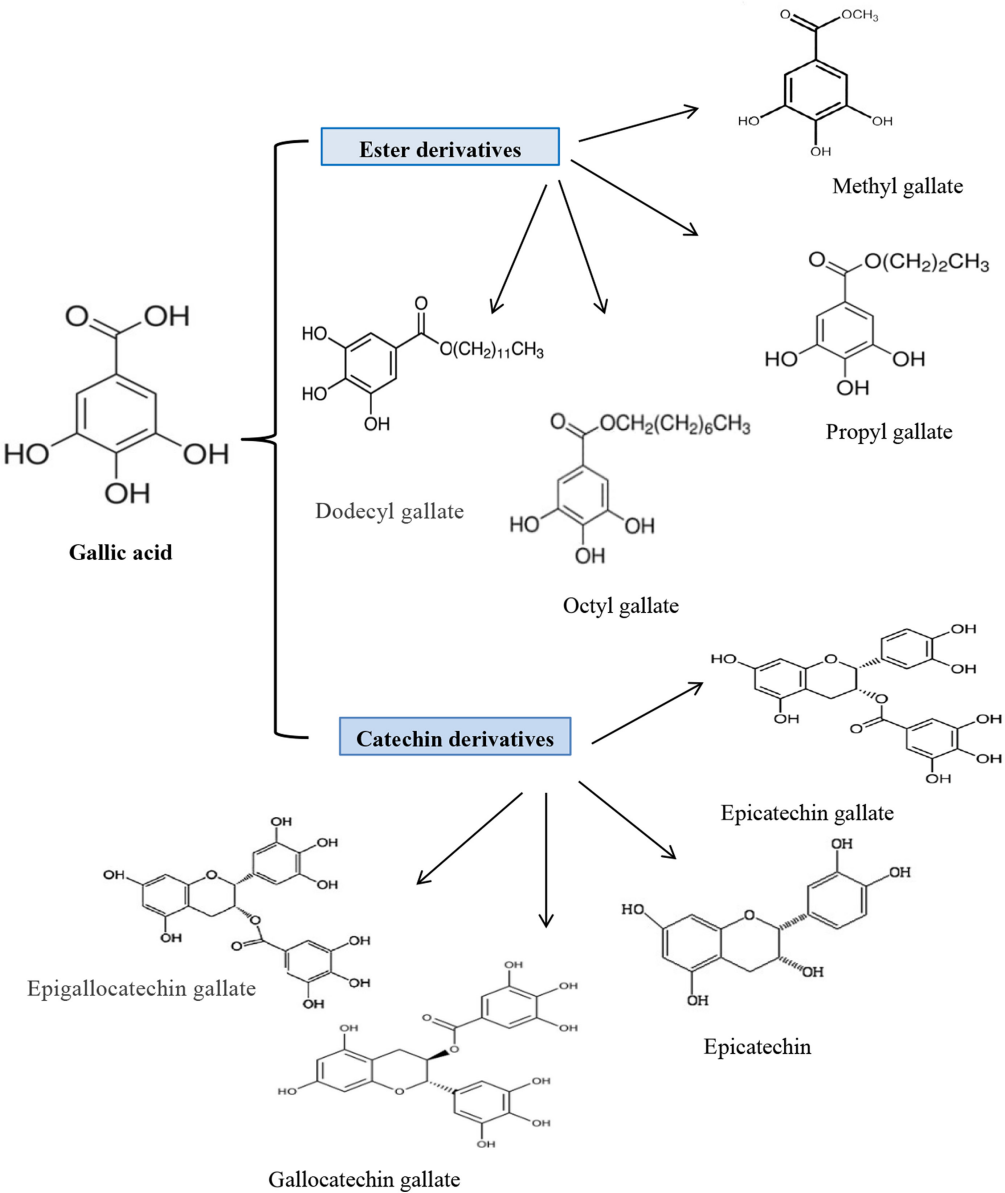
Chemical structure of GA and its derivatives.

GA esters are also present in many plants and are identified as gallates. GA derivatives exist in two forms: ester and catechins (Figure 1). Its ester derivatives are in the form of alkyl esters, such as methyl gallate, propyl gallate, and octyl gallate, and the most important catechin derivatives are epicatechin gallate, epicatechin, and gallocatechin gallate (Badhani et al., 2015; Yang, Zhang, et al., 2020). They have antioxidant capacity against active species of oxygen and nitrogen such as hydrogen peroxide (H_2_O_2_), hydroxyl (HO^·^), peroxyl (ROO^·^), and azide radicals. These ester derivatives, like propyl gallate, octyl gallate, and lauryl gallate, act as scavengers of ROS and are used in the packaging of food and processed foods to prevent oxidative spoilage (Badhani et al., 2015). In addition, GA esters can increase the bioavailability of drug compounds by acting on cellular transporters such as uridine diphosphate‐glucuronosyl transferase 2B (UGT2B) (Choubey et al., 2015).

Fuiza et al. investigated the effect of GA esters on the human cervical adenocarcinoma cells (HeLa line). They reported the size, substituted groups of hydroxyl rings, the number of carbons in the alkyl chain, and lipophilicity of the structure have a decisive effect on the anticancer activity of the GA. The results showed compounds containing propyl esters have a more inhibitory effect on Hela cells compared to derivatives containing methyl and octyl groups. These results probably depend on the balance between the drug's hydrophobicity and hydrophilicity. Because for a drug to exert its effect in the cell, it must pass through two environments: the membrane (hydrophobic environment) and the cytoplasm (hydrophilic environment). Considering that the degree of hydrophobicity of ester derivatives depends on the number of carbons in the alkyl chain, therefore, compounds with moderate hydrophobicity and hydrophilicity (moderate carbon chain) can exert more toxic effects on the cells (Fiuza et al., 2004). Based on quantitative structure–activity relationship (QSAR) models, Bouarab‐Chibane et al. (2019) also reported the antibacterial activity of polyphenols depends on their lipophilicity, electronic properties, and electrical charge which leads to the interaction of the compound with the bacterial surface. Another group of researchers has shown that most of the time, GA derivatives have more antibacterial activity compared to GA itself. This can be due to the more lipophilicity of derivatives, which increases their ability to pass through the cell membrane (Choińska et al., 2021).

Consumption of GA worldwide is about several thousand tons per year, while its natural production is limited and does not meet this need. At present, GA can be produced industrially by the oxidation of natural gallotanins such as tannic acids (Aguilar‐Zárate et al., 2015). Acids and alkalines can hydrolyze tannic acid to produce GA. One of the common methods of precipitating GA from aqueous solutions is the use of concentrated sulfuric acid. But because of its disadvantages such as high cost, low efficiency of acid hydrolysis, and the production of large toxic effluents, the use of new methods based on biocompatible compounds is essential. Nowadays, microorganisms are considered a suitable factor for the production of GA due to their ability to hydrolyze tannic acid by producing the enzyme tannase that catalyzes the hydrolysis of ester bonds present in gallotannins (Brewer, 2011; Moghtaderi et al., 2018). Fungi such as *Fusaria*, *Aspergilii*, *Trichoderma*, and *Penicilii*, and bacteria such as *Lactobacillus* sp., *Bacillus*, *Corynebacterium* sp., *Serratia* sp., *Streptococcus*, *Pseudomonas*, *Enterococcus*, have been reported as tannase producing microorganisms (Aguilar‐Zárate et al., 2015).

## 3 THE PHARMACOKINETICS AND BIOAVAILABILITY OF GA

A group of researchers has found that GA after entering the body spreads rapidly to all tissues, with the highest distribution being in the kidney, heart, spleen, liver, and lungs. While another group believes that GA is distributed only in the liver and kidneys (Chen et al., 2018). Researchers have shown that GA is extensively metabolized to various compounds after entering the gastrointestinal tract; for example, Barnes et al. examined the uptake and metabolism of mango (as a source of GA) in healthy volunteers for 10 days. They identified seven metabolites of GA in their urine, including pyrogallol‐1‐O‐glucuronide, 4‐OMeGA, 4‐ OMeGA‐3‐O‐sulfate, pyrogallol‐O‐sulfate, deoxy pyrogallol‐Osulfate, and O‐methylpyrogallol‐O‐sulfate (Barnes et al., 2016). In fact, various GA metabolites have been identified in the body, such as ethyl gallate, 3‐dihydrochemical acid, and different type's galloyl‐glucose derivatives. Also, GA can also be converted to syringic acid via the methylation of oxygen (Bai et al., 2021). Methylated, sulfated, and glucoside metabolites are major constituents of GA. Konishi et al. examined the intestinal absorption of GA in rats after oral administration. The rats received a single 100 μmol/L of GA dose. The drug was absorbed at a slow rate and GA and 4‐O‐methyl GA (4OMGA) were observed in the serum (Konishi et al., 2004). 4OMGA was identified as the major metabolite of GA, tannic acid, propyl gallate, and lauryl gallate in the urine of rats and rabbits (Moghtaderi et al., 2018). In humans, 4OMGA is also the first metabolite of GA produced in plasma and urine. Studies in humans have shown that GA is broken down and absorbed in the gastrointestinal tract. It was metabolized in the liver and eventually excreted by the kidneys. After oral administration, about 70% of it was absorbed and then excreted as 4OMGA in the urine. GA has a high absorption compared to hundreds of food polyphenols and as a source of natural product, its health effects have been confirmed in humans (Gulcin et al., 2019; Manach et al., 2005).

Studies on the bioavailability of GA have been performed in animals and humans. It has been reported that rapid metabolism and excretion of GA reduce its effectiveness. Polyphenol compounds are generally metabolized rapidly in the gut, they are rapidly absorbed and excreted in the body, resulting in low bioavailability (Crozier et al., 2009; Laddomada et al., 2015; Zhang, McClements, et al., 2020). To solve this problem, the biopolymer‐based delivery system is designed using proteins, polysaccharides, and phospholipids to increase physicochemical properties, stability, and the absorption of polyphenols through the intestine and reducing their excretion rate, thereby increasing their bioavailability in the body. This method has been able to greatly increase the effectiveness of polyphenols in the food industry and biomedical applications (Bhattacharyya et al., 2013; Zhang, McClements, et al., 2020). Nanotechnology is also an ideal transport system for polyphenolic compounds such as GA. Nanoformulation drug delivery systems such as micelles, emulsions, niosomes, polymers, and metal nanoparticles can overcome the limitations of using polyphenols, protect active substances, and control their release. However, if nanoparticles are used, their safety should be thoroughly evaluated after entering the body (Yang, Dong, et al., 2020).

In vivo and in vitro studies have shown that the toxicity of GA is relatively weak, so it is effective and safe in low concentrations in most cells, while in high concentrations it is slightly toxic (Bai et al., 2021). For example, acute GA toxicity was observed in albino mice when the LD50 of GA was above 2000 mg/kg. Hematological examinations, gross necropsy, and histopathological studies at dose sub‐acute toxicity of GA (≤ 900 mg/kg/day) for 28 days did not cause any significant changes in blood homeostasis, tissue histology, and morphological and behavioral parameters of the male and female albino mice, and confirmed the safety of GA in mice (Variya et al., 2019). Another group of researchers investigated the effect of GA on neuronal cells. They reported that concentrations of 5–50 μM GA did not affect BV‐2 and Nerve‐2A cell survival, while concentrations above 100 μM were toxic to the cells (Kim et al., 2011). Ghafor et al. investigated different concentrations (0–500 μg/mL) of GA‐loaded graphene oxide (GO) (GAGO) on zebrafish embryogenesis for 24‐96 h. The results showed that low concentrations of GAGO (0–150 μg/mL) had no toxic effect on cells at all times and are completely safe but at higher concentrations, mortality significantly increased, hatching rate delayed, and heart rate decreased (Abdul Ghafor, 2020). Other researchers reported the concentration of 75 μM GA caused apoptosis in the A549 cell line via the PI3K/Akt Pathway, while this concentration of GA did not have a destructive effect on the normal lung fibroblast cells (W‐26 cell line) (Ko et al., 2022). Devl et al. compared the concentration of 5–100 μg/mL of GA on HCT150 cells and normal human lymphocyte cells. Their results showed that GA caused severe DNA damage in the colon cancer cells and had potent anticancer effects on these cells, while no toxic effect was observed on lymphocytes (Devi et al., 2015). Therefore, the conducted studies show that GA is a safe agent for normal cells, although its dosage should be considered.

## 4 APPLICATIONS OF GA AND ITS DERIVATIVES

**FIGURE 2:**
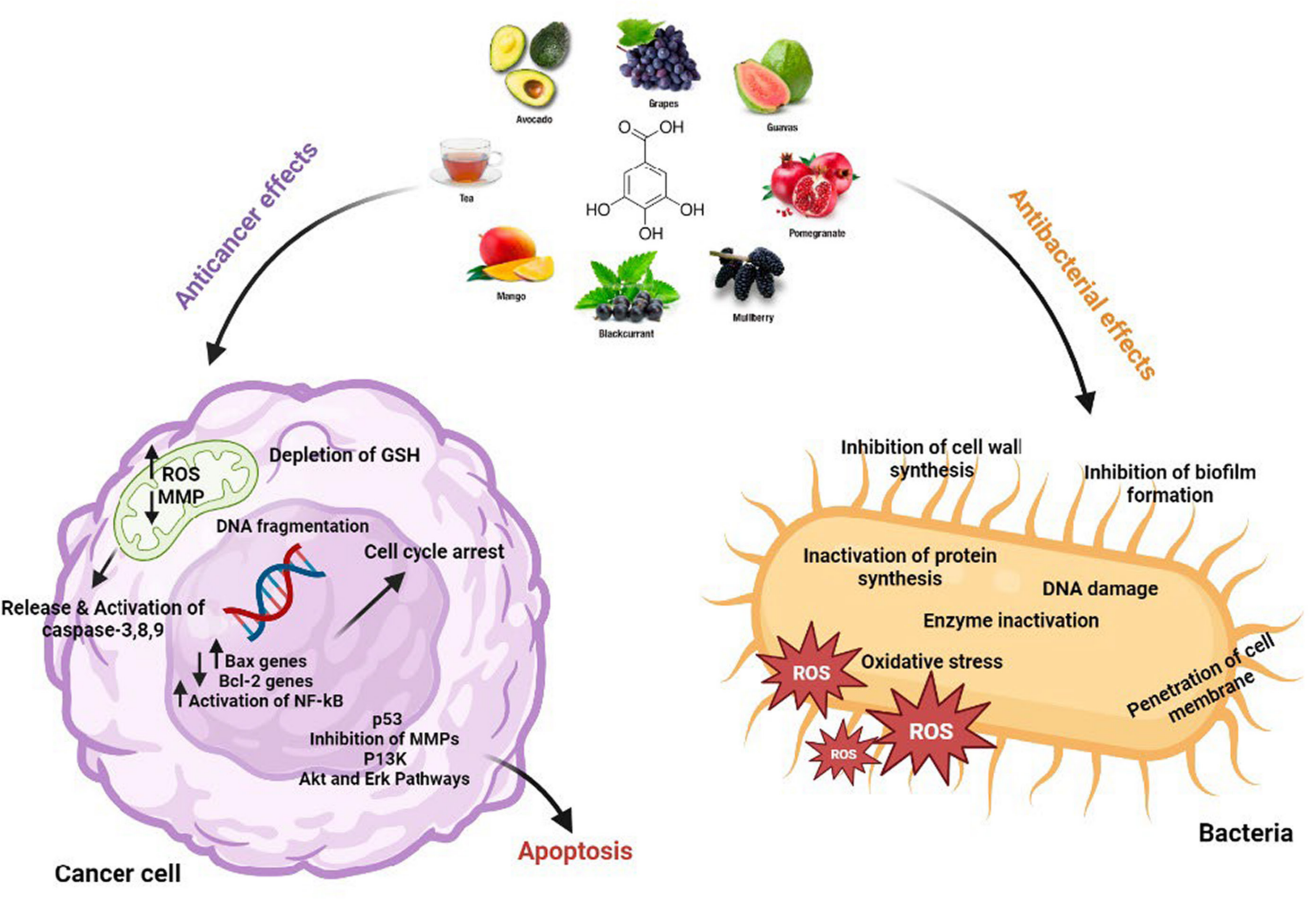
Schematic description of anticancer and antibacterial mechanisms of gallic acid.

The therapeutic effects of GA are widespread. It can act as an antibacterial, anticancer, antiviral, antioxidant, antiallergy, and antiinflammatory agent (Figure 2). It is also effective in cardiovascular diseases, and metabolic diseases such as diabetes, obesity, and degenerative diseases (Gao et al., 2019). In the following sections, we describe the latest findings about the antibacterial and anticancer activity of GA and its derivatives.

### 4.1 Antibacterial activity of GA

GA as an active phenolic compound in plants has a wide range of antimicrobial activities against humans and plant pathogens as well as pathogenic yeasts in humans (Table 1) (Choubey et al., 2018). Researchers have shown that GA not only has antimicrobial effects against various bacteria but also increases the effectiveness of antibacterial compounds such as ciprofloxacin, erythromycin, norfloxacin, oxacillin, ampicillin, gentamicin, and penicillin via synergistic function (Rajamanickam et al., 2019).

**TABLE 1:** The antimicrobial activity of GA.

Compounds	Strains	Functions	References
GA	*Escherichia coli*	Blocked the catalase activity, inhibition of biofilm formation by the effect on the pgaABCD genes expression	Kang, Li, et al. (2018), Wang et al. (2018)
GA	*Klebsiella pneumoniae*	Disrupted membrane integrity, inhibited the growth and production of capsular polysaccharide	Khorsandi et al. (2021)
GA	*Staphylococcus aureus*	Inhibited the biofilm formation by the effect on the expression of the ica operon	Liu et al. (2017)
GA	*Shigella flexneri*	In the destroyed cell, the bacterial morphology inhibited biofilm formation by the effect on the expression of the mdoH gene and the OpgH protein	Kang, Liu, et al. (2018)
Octyl gallate	*E. coli and S. aureus*	Destroying the cell wall, Penetration the cell, interacting with DNA, damaging the activity of the respiratory chain, increasing the production of ROS	Shi et al. (2021)
GA‐grafted‐chitosans + ampicillin penicillin oxacillin	MRSA	Had synergic effects on bacteria, induced loss of membrane integrity, and released intracellular components	Lee et al. (2014)
GA+ hydroxytyrosol	*E. coli S. pyogenes*, *K. Pneumoniae*, *S. aureus*	Inhibited the growth of bacteria and had a synergistic effect against all four strains	Tafesh et al. (2011)
GA+ ceftiofur	*S. Typhimurium*	This compound had an additive effect on bacteria, inhibited the growth of plankton and bacterial biofilm, changed the morphology of bacteria, and inhibited the swimming and swarming motilities of bacterial	Hossain, Park, Lee, et al. (2020)
GA+ thiamphenicol	*E. coli*	Altered bacterial morphology, synergistically inhibited the growth of plankton and bacterial biofilms	Hossain, Park, Park, et al. (2020)
Au‐NP‐ GA	*P. shigelloides S. flexneri*	Destruction of membrane surface macromolecules	Daduang et al. (2016)
GA‐ loaded – ZnO NPs	MRSA	Had strong antioxidant and antibiotic effects	Lee et al. (2017)
GAGO	MRSA	The nanoformulation significantly increased the antibacterial activity of GA	Shamsi et al. (2018)
GA‐g‐chitin‐glucan complex	*E. coli B. subtilis*	Had stronger antibacterial properties compared to the unmodified chitin‐glucan complex	Singh et al. (2019)

Abbreviations: GA‐g‐chitin‐glucan, GA grafted chitin‐glucan complex; *GAGO*, GA Loaded Graphene Oxide; *P. shigelloides*, *Plesiomonas shigelloides*; *S. flexneri*, *Shigella Flexner*.

Borges et al. have shown the antibacterial activity of GA against *E. coli*, *Pseudomonas aeruginosa* (*P. aeruginosa*), *S. aureus*, and *Listeria monocytogenes* (*L. monocytogenes*). GA caused membrane pores in the bacteria and led to irreversible changes in bacteria by altering membrane permeability, hydrophobicity, and physicochemical properties of bacteria (Borges et al., 2013). Rangel et al. have proposed that ester derivatives of GA reduce drug resistance in *Saccharomyces cerevisiae* (*S. cerevisiae*) through inhibition of ABC transporter Pdr5p. They proved that ester derivatives with 8–16 carbon in the side chain have a strong inhibitory effect on transporter Pdr5p (Pereira Rangel et al., 2010). GA‐grafted‐Chitosan inhibited the growth of gram‐positive and gram‐negative bacteria by destroying cell membranes and increasing their permeability (Choubey et al., 2018). A comparative study of the antibacterial effects of chlorogenic acid and its metabolites showed that GA has a strong antibacterial activity among other metabolites, especially against Meticillin‐Sensitive *S. aureus* (MSSA) and Methicillin‐resistant *S. aureus* (MRSA). GA was found to destroy membrane adhesions and increase membrane permeability, leading to increased bacterial fluid conductivity (Lu et al., 2021). Another study showed GA effectively can inhibit *Streptococcus mutans (S. mutans)* biofilm formation (de Lima Pimenta et al., 2013). Passos et al. have studied GA and ethyl gallate isolated from the fruit and seeds of *Libidibia ferrea* against *Streptococcus mutans*. The results showed the compounds reduced the expression of gtfB, gtfC, and gtfD genes in biofilm. In addition, they inhibited adhesion, and reduced exopolysaccharide, biomass, and bacterial microclines in the biofilms (Passos et al., 2021). Kang et al. have shown that GA inhibits the growth of planktonic *S. flexneri* by reducing cell viability, and membrane adhesion, and causing morphological changes. It also effectively inhibited the formation of biofilms in bacteria by reducing the expression of the *mdoH* gene and the OpgH protein that stopped the synthesis of polysaccharides within the biofilm, thus preventing the formation and stability of biofilms (Kang, Liu, et al., 2018) (Pereira Rangel et al., 2010). GA inhibited biofilm growth in *E. coli* by regulating *pgaABCD* gene expression and reducing the synthesis of polysaccharides, proteins, and DNA in the biofilm. Thus, GA has been proposed as a new natural and safe approach to control biofilm‐related infections (Kang, Li, et al., 2018). Bisignano et al. have studied the effect of pistachio polyphenols such as GA on 44 *S. aureus* clinical isolates (9 MRSA) derived from skin infections and surgery. The results showed the significant bactericidal effects of GA on *S. aureus* (Bisignano et al., 2013). Another group of researchers studied phenolic compounds (caffeic, ellagic, and GA, kaempferol, quercetin, and rutin) derived from plants on skin infections including *Staphylococcus epidermidis* (*S. epidermidis*), *S. aureus*, and *K. pneumonia*. The results showed that the antibacterial effects of GA against bacteria were greater than other compounds. The minimum bactericidal concentration (MBC) of GA against the investigated bacteria was finally 0.04 mg/mL, at this concentration, the survival of animal fibroblast cells was more than 70%. Therefore, GA can be used as a safe compound against these bacteria in infectious wounds without harming fibroblast cells (Pinho et al., 2014).

Birhanu et al. reported methyl gallate, a GA derivative inhibited invasion, adhesion, and intracellular survival of *Salmonella Typhimurium* (S. *Typhimurium*) (Birhanu et al., 2018). Hydroxyl groups in GA derivatives can inactivate bacterial proteins such as enzymes, receptors, ion channels, and carriers by binding with them and forming ionic and protonic bonds (Buchmann et al., 2022; Hossain, Park, Park, et al., 2020). Another group investigated the antibacterial action of alkyl gallates on *E. coli* and *S. aureus*. Studies have shown that alkyl chain length plays a major role in the activity of GA derivatives, so octyl gallate showed strong antibacterial activity. Octyl gallate had a bactericidal function in both bacteria by destroying the cell wall, damaging the respiratory chain's activity, and increasing ROS production (Shi et al., 2021).

According to the results obtained from the action of GA on bacteria, it can be used to promote antimicrobial technologies in the field of treatment and the food industry.

#### 4.1.1 Antibacterial activity of GA in combination with other antibacterial agents

Due to the widespread resistance of bacteria to existing antibiotics, combination therapy is one of the most effective ways to increase the effectiveness of existing antibiotics. In addition, combination therapy reduces the spread of drug resistance due to reduced antibiotic doses (Buchmann et al., 2022). Hossain et al. investigated the combined effect of GA and its derivatives with traditional antibiotics on *E. coli*. The results showed the combination of GA with Thiamphenicol had a synergistic effect on *E. coli*. GA with cefotaxime, marbofloxacin, and amphetamine had an additive effect. Subsequent analysis showed GA‐ampicillin inhibited the growth and viability of biofilm in *E. coli* in humans and animals (Hossain, Park, Park, et al., 2020).

Another group of researchers studied the combined effect of GA ethyl ester with conventional antibiotics, such as fusidic acid, minocycline, and rifampicin, against *S. aureus* strains. The results showed that the combination of GA ethyl ester with all antibiotics had an additive effect against the studied strains (Kyaw et al., 2012). Another study revealed that phenols isolated from the alcoholic extract of Thai mango kernel including GA and methyl gallate had a synergistic effect with penicillin G against MRSA clinical isolates and increased the antibacterial function of this antibiotic in the isolates by bacteriostatic effect (Jiamboonsri et al., 2011). Gutiérrez‐Fernández et al. studied the antibacterial effects of three natural phenols (thymol, carvacrol, and GA), and two synthetic phenols (octyl gallate, and butylated hydroxyanisole) alone and in combination against resistant *Enterococcus faecalis* (*E. faecalis*) dairy isolates. The results noted that GA in combination with octyl gallate is effective in controlling bacteria in dairy products in such a way that their combined effect significantly reduced MIC of compounds compared to their use alone (Gutiérrez‐Fernández et al., 2013). Lima et al. demonstrated the synergistic effect of GA with norfloxacin and gentamicin against *S. aureus*. The MIC of norfloxacin alone against bacteria was 156.3 μg/mL, which was reduced to 49.21 μg/mL in combination therapy. Therefore, the combined treatment led to a reduction of MIC up to 31.48%. The MIC of gentamicin alone also decreased from 49.21 μg/mL to 2.44 μg/mL (4.95% reduction compared to control) (Lima et al., 2016). Wang et al. investigated the effect of GA in the presence of UV‐A light against *E. coli*. GA alone had a mild antibacterial effect. UV‐A light with a wavelength of 360 nm and average intensity of 3425 μW/cm^2^ for 30 min enhanced the effectiveness of GA. Initially, UV‐A light increased the uptake of GA into the bacteria. The interaction of GA and UV increased the production of ROS, inhibited the activity of superoxide dismutase, and caused oxidative stress in the bacteria by unbalancing the redox state of the cells. Oxidative stress caused the death of bacteria by affecting macromolecules, metabolism, and bacterial structure (Wang et al., 2017).

#### 4.1.2 Antimicrobial activity of GA in nanoparticle formulation

Nanoparticles are a suitable drug transmitter for the treatment of infections caused by resistant bacteria because the possibility of bacterial resistance to nanoparticles is very low. In addition, they increase the stability and biocompatibility of drug compounds and targeted drug delivery (Mazzotta et al., 2021; Shamsi et al., 2018). Sun et al. designed a nanocomposite containing chitosan‐copper‐GA and then used it as an antibacterial dressing on wounds infected with *S. aureus*. In addition to the positive effect on the removal of bacteria, the designed composition had no side effects on the normal cells. Its effective application makes its use effective in the field of biomedicine (Sun et al., 2021). Hoyo et al. designed nano‐hybrid‐coated contact lenses containing ZnO NPs‐chitosan‐GA. The synthesized nanocomposite had antioxidant effects and increased the wettability of lenses. It also had a high antibacterial effect against *S. aureus*, which is directly related to problems with contact lenses such as microbial‐associated keratitis, peripheral ulcer, and acute red eye (Hoyo et al., 2019). Another study showed that the conjugation of GA with Au‐NPs increased the antibacterial effects of GA against foodborne pathogens. FTIR studies have shown that Au‐NP‐GA leads to changes in lipids, nucleic acids, and proteins in bacterial membranes (Daduang et al., 2016). Shamsi et al. evaluated the antibacterial effects of GAGO as a popular graphene‐based compound on MRSA and MSSA. The results showed the preparation of this nanoformulation significantly increased the antibacterial efficacy of GA against MRSA compared to GA alone. It is suggested that this compound can be a suitable antibacterial agent against multi‐drug‐resistant bacteria (Shamsi et al., 2022). Another study showed that the effectiveness of GAGO against MSSA and MRSA was comparable to the first 2 hours of exposure of bacteria to GA and GO alone. They reported that its efficacy on bacteria at concentrations of ≥150 μg/mL is comparable to cefoxitin. Moreover, this nanoformulation increased the biocompatibility of GA in physiological environments and GAGO had little toxicity in 3T3 murine fibroblast cells and zebrafish embryos cells at all investigated times (24, 48, 72 h) compared to GA alone (Shamsi et al., 2022). Another group of researchers designed liposomes loaded with GA (GA‐LIP) and GA‐LIP decorated with Lactoferrin (LF‐GA‐LIP). LF‐ GA‐ LIP showed stronger antibacterial activity than GA‐LIP against *E. coli* and *S. aureus* and it had a slower release rate in a simulated digestive system. Therefore LF‐GA‐LIP was introduced as a suitable transport system in the food industry (Zhang, Pu, et al., 2019). Li et al. synthesized Ag‐NPs coated with GA and studied their antibacterial effects on *E. coli*, and *S. aureus*. The results showed high activity of GA‐AgNPs which had very little toxicity on normal cells. It was suggested that the GA‐AgNPs could be used in medical devices, pharmaceutical applications, and silver‐based wound care products to eliminate bacterial infections, although their safety in animals should be examined more closely (Li et al., 2015). The other group synthesized modified Ag‐NPs using GA and chitosan (GC‐AgNps) by ultrasonication and investigated their antibacterial effects on *E. coli*. In addition to being a quick, easy, and cost‐effective method, the synthesized combination showed strong antibacterial results. GC‐AgNPs bind to the peptidoglycan membrane destroyed the lipopolysaccharide barrier, and penetrated the membrane protoplasm, also increasing ROS production due to contact with intracellular components and scavengers' depletion (Guzmán et al., 2019).

Overall, the results show that the use of GA as nanoparticles increases its efficacy, although the performance and safety of GA‐carrying nanoparticles in vitro and in vivo need to be evaluated. Because polyphenols such as GA as a natural substance should be consumed for a long time and considering that different types of nanoparticles can have a negative effect on the lungs, liver, kidneys, brain, and sexual organs, their use as GA carriers should be considered and its side effects should be minimized (Carvalho et al., 2018; Lin et al., 2022).

The antibacterial effects of GA alone, in combination with other drugs, and as a nanoparticle formulation against different types of bacteria are summarized in Table 1.

#### 4.1.3 Anticancer activity of gallic acid

GA and its derivatives can be used as anticancer agents. Several studies have demonstrated that GA and its derivatives have an anticancer effect in cancers such as prostate cancer, melanoma, leukemia, oral cancer, colon cancer, lymphoma, and breast cancer cells. The following sections described the molecular actions initiated by GA and its derivatives on various cancer cells (Subramanian et al., 2015).

##### Anticancer activity on prostate cancer cells

In DU145 prostate cancer cells, GA was the primary anticancer compound that suppressed the cells' growth. GA reduces the cell survival of DU145 cells by generating ROS and mitochondria‐mediated apoptosis. Also, GA leads to cell cycle arrest at the G2/M phases by activating Chk1 and Chk2 and inactivating Cdc25C and Cdc2 (Chen et al., 2009). The autoxidation of GA is effective on malignant prostate cells and increases ROS levels. Loss of mitochondrial potential and release of cytochrome c from the mitochondria to the cytosol led to the activation of caspases 3, 8, and 9. In prostate cancer cells, GA leads to dose‐dependent apoptosis (Russell Jr et al., 2012).

A study investigated the antitumor effect of GA on PC3 prostate cancer cells. GA can induce DNA damage and recruits several DNA repair genes (Liu et al., 2015). Also, it was investigated that GA can eliminate the migration and invasion of PC3 human prostate cancer cells (Meng, 2011). There was inhibition of JNK, PKC, p38, and P13K/AKT signaling pathways in PC3 cells treated with GA and it finally blocked MMP‐2 and ‐9 in these cells (Subramanian et al., 2015).

##### Anticancer activity on melanoma cells

The GA showed significant cell proliferation inhibition and apoptosis induction on A375S2 human melanoma cells (Kondo et al., 2009). After GA treatment, the proliferation of cells decreased in a dose and timely manner. The apoptosis molecular mechanism is done by the downregulation of antiapoptotic Bcl‐2 and upregulation of the proapoptotic B‐cell lymphoma 2 (Bcl‐Fp132) associated X protein (Bax). By decreasing the mitochondrial membrane potential in a time‐dependent manner, GA induced the release of cytochrome c, promoting the activation of caspase 9 and 3, and finally apoptosis. Also, GA induced the expression of endonuclease G (Endo G) and apoptosis‐inducing factors (AIF) (Lo et al., 2011). A study showed the influence of GA on the gene expression and protein levels of MMPs and in vitro migration of melanoma cells; as a result, the treatment of the GA leads to a reduction in the MMPs signal pathway and mRNA levels in A375S2 cells. Thus, GA acted as an antimetastatic agent. In addition, this was included in the Ras, and p‐ERK signaling pathways, causing the suppression of MMP‐2 in A375S2 melanoma cells (Subramanian et al., 2015).

##### Anticancer activity on colon cancer cells

A study investigated the anticancer property of GA against COLO 205 cells of colon cancer. GA treatment leads to the fragmentation of DNA. Morphological changes, especially observing apoptotic bodies, indicated that GA caused apoptosis (Yoshioka et al., 2000). Another study mentioned an antitumor effect of GA on HCT‐15 colon cancer cells. GA decreased the survival of colon cancer cells in a dose‐dependent manner. Cell contraction, cell rounding, and separation from the substrate were notable changes in GA‐treated cells compared to the control (Devi et al., 2014). Generally, GA derivatives treatment reduces cell viability; also, the antioxidant influence and structure of GA derivates showed a similar manner (Khaledi et al., 2011).

##### Anticancer activity on leukemia

Several studies showed the anticancer property of GA against HL 60 and HL‐60RG promyelocytic leukemia and also K562 human leukemia cells through cell cycle arrest at the G0/G1 phase. Inhibition of ribonucleotide reductase, DNA damage, and fragmentation, the release of cytochrome c, upregulation Bcl‐2 protein, AIF & Endo G, activation of caspase 4, 9, and 3, inhibition of BCR/ABL tyrosine kinase, activation of NF‐κB, increasing levels of Bax & Fas ligand and p53 have been reported as another anticancer effect of GA. (Chandramohan Reddy et al., 2012; Htay et al., 2002; Locatelli et al., 2008; Madlener et al., 2007; Moriwaki et al., 1991; Yeh et al., 2011) (Table 2).

**TABLE 2:** The anticancer activity of GA.

Compounds	Cancer	Function	References
GA	Colon cancer (Male albino Wistar rats)	Increased superoxide dismutase, catalase, glutathione reductase, and glutathione peroxidase activity	Giftson et al. (2010)
GA	Nonsmall‐cell lung cancer (A549 and NCI‐H1299 cell lines)	Inhibited proliferation and elevated apoptosis in cells by suppression of EGFR and reduced CARM1‐PELP1 complex formation	Wang and Bao (2020)
GA	Gastric adenocarcinoma cells	Induced apoptosis by up‐regulation of Fas, FasL, and DR5 expression	Tsai (2018)
	AGS cells (ATCC CRL 1739).		
GA	Bladder cancer (T24 cell line)	Inhibited the cell proliferation by disrupting PI3K/Akt/NF‐kB signaling pathways	Zeng et al. (2020)
GA	Leukemia sensitive and its resistant sublines (HL60 cell HL60/VINC HL60/MX2)	Altered cell cycle distribution and increased cell population in sub‐G1	Maruszewska and Tarasiuk (2019)
		Modulated ROS production in cells in a time‐ and dose‐dependent manner	
GA + paclitaxel carboplatin	Breast cancer (MCF‐7 cell line)	Cell cycle arrest at the G2/M phase, the minimum concentration of GA increased the induction of apoptosis by drugs, and triplet combination upregulated the expression of P53, Bax, and CASP‐3.	Aborehab et al. (2021)
GA+ Paclitaxel	Cervical (HeLa cells)	Increased apoptosis by upregulation of p53 and caspase 3	Aborehab and Osama (2019)
GA+ Caffeic acid	Breast cancer (MCF‐7 cells)	Activation of apoptosis signaling pathways by regulating the expression of P53, Mcl‐1, and P21	Rezaei‐Seresht et al. (2019)
GA+ low‐level laser	Melanoma and breast cancer (A375, and MDA‐MB‐231 cells)	Generated ROS and inducted apoptosis and ferroptosis	Khorsandi et al. (2020)
GA+ Cisplatin	Small cell lung cancer (H446 cells)	Increased cisplatin effect by producing ROS, increased Bax, Apaf‐1, DIABLO, and p53 expression, decreased XIAP expression, and MMP degradation	Wang et al. (2016)
GA+ Temozolomide	Human glioma cell (U87MG cells)	Increased the performance of the drug by reducing of Bcl‐2 expression and Akt activation, and activation of the p38‐MAPK pathway	Yang et al. (2022)
GA‐Gold NPs + radiotherapy	Human glioma cells (U251 cells)	Inhibited cell viability, increased radiotherapy function, induced apoptosis by increasing BAX expression, downregulated Bcl‐2 expression, and stopped cell cycle in S and G2/M phases.	Jing et al. (2021)
Graphene Oxide‐GA	Liver cancer cells (HepG2 cells)	Inhibited the growth of cancer cells without damaging normal cells	Dorniani et al. (2016)
Conjugated of the GA with PAMAM dendrimers	Human colorectal carcinoma (HCT 116 cells)	Synthesized particles increased GA uptake, inhibited cell proliferation and their colonogenic ability, decreased cancer cell migration by reducing MMP expression	Priyadarshi et al. (2021)
		Promoted apoptosis in cancer cells by inhibiting NF‐κB activation	
GA nanoparticles coated with alginate‐chitosan	Breast cancer cells (T47D cells)	Had strong cytotoxicity in cancer cells	Arsianti et al. (2020)

##### Anticancer activity on cervical cancer cells

HeLa cervical cancer cells treated by GA showed depletion in GSH, decrease in mitochondrial membrane potential, downregulation of the EGFR, Erk/p‐Erk, and Akt/p‐Akt signaling pathways, activation of caspases and poly (ADP) ribose polymerase cleavage and cell cycle arrest in G1 phase (You et al., 2010; You & Park, 2011; Zhao & Hu, 2013).

**FIGURE 3:**
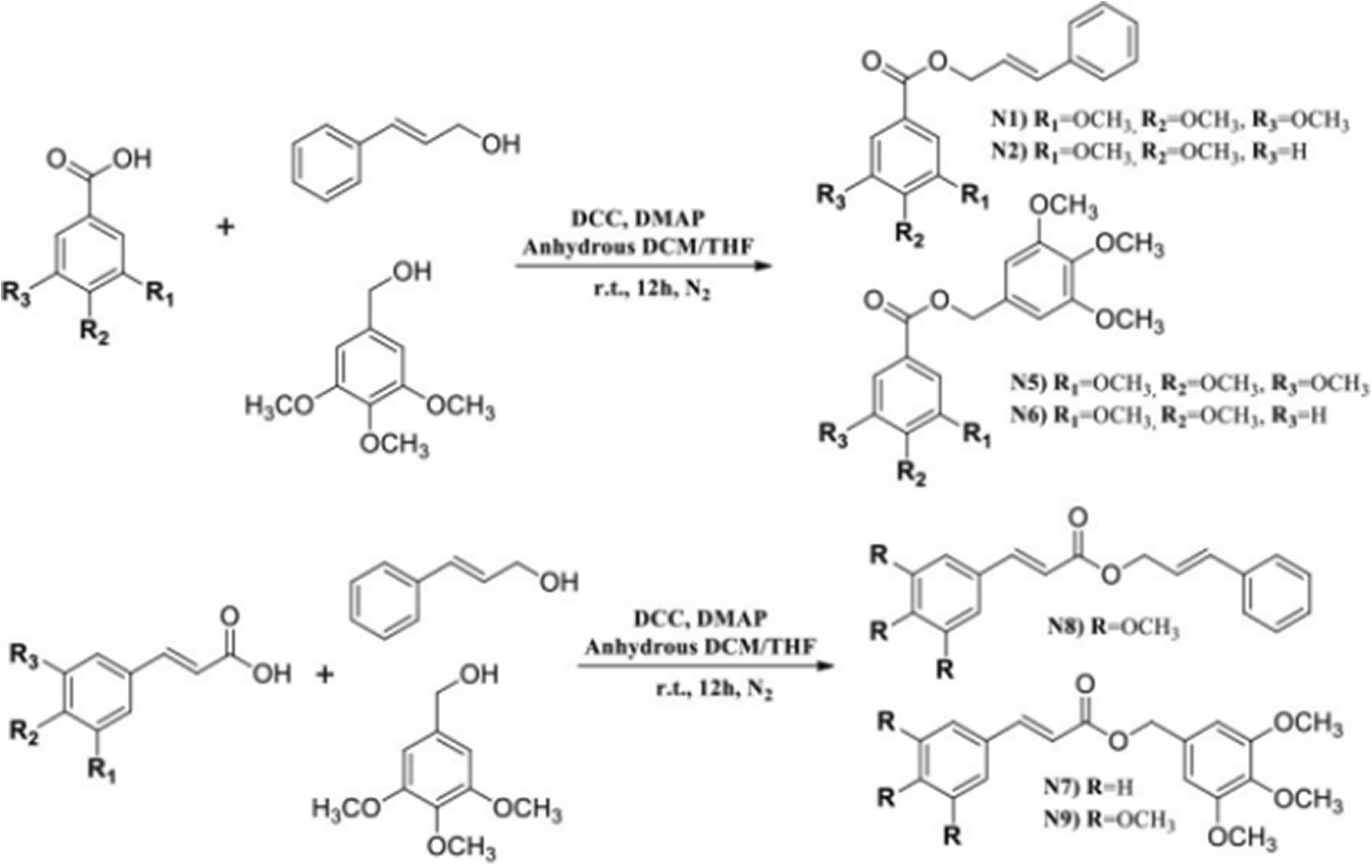
Gallate analogs.

Another group of scientists synthesized nine chimeric gallate‐cinnamate derivatives using QSAR (Figure 3). Then their cytotoxicity activity was investigated on HeLa cells and nontumorigenic HaCaT cells. QSAR model showed the importance of structure, attachment of specific groups to phenolic rings, lipophilicity, size, and electronic charge of gallate‐cinnamate derivatives to inhibit the growth of HeLa cells. Among the designed models, 3,4,5‐trimethoxybenzyl 3,4‐dimethoxy benzoate (N6), 3,4,5‐trimethoxybenzyl 3,4,5‐trimethoxybenzoate (N5), cinnamyl 3,4,5‐trimethoxybenzoate (N1), 3,4,5‐trimethoxybenzyl 3,4,5‐trimethoxycinnamate(N9) had the more effect on inhibiting Hela cells (with IC_50ExpHeLa_ 7.26–11.95 μM), while they had no toxic effect on HaCaT cells. Finally, molecular docking studies showed that the binding of compounds to tubulins inhibits the growth of cancer cells (Nolasco‐Quintana et al., 2023).

#### 4.1.4 Anticancer activity of GA in combination with anticancer agents

The anticancer mechanism of GA alone or in combination with cisplatin was found in nonsmall cell‐lung cancer (NSCLC). The studies show that GA prompted the apoptosis of NSCLC A549 cells and restrained the proliferation in the dose‐ and time‐dependent manners with downregulating Bcl 2 and upregulating B‐cell lymphoma 2 (Bcl‐Fp132)‐associated X protein (Bax). Also, the findings showed that GA increases Bax expression and inhibits Bcl 2 expression which finally increases the anticancer property of cisplatin and induces cell apoptosis. Furthermore, these studies indicated that GA alone has anticancer effects on NSCLC A549 cells and increases cisplatin's anticancer effects by inducing the JAK/STAT3 signaling pathway and downstream apoptotic molecules (Zhang, Ma, et al., 2019).

Khorsandi et al. found that pretreatment with low‐level lasers and then the GA treatment on breast cancer and melanoma cells induce more cell death in comparison to GA alone. They indicated that ROS production in cells treated with low‐level lasers and then GA was more than in cells treated only with GA. It is noted that the combination of low‐level laser irradiation and then GA may increase cell death through cell death apoptosis and ferroptosis mechanisms compared to GA alone (Khorsandi et al., 2020).

Paclitaxel/carboplatin is one of the most common drugs in treating cervical cancer. Furthermore, chemotherapeutic drugs are very beneficial, but intensive side effects and the development of drug resistance limit these drugs' use. Using natural products with anticancer activity may help to overcome these problems. These findings indicated that GA increases the paclitaxel property. A combination of paclitaxel and GA could display a promising replacement with lower side effects for paclitaxel/carboplatin combination in cervical cancer treatment (Aborehab & Osama, 2019).

GA and curcumin (Cur) are two natural phenolic compounds and demonstrated their anticancer properties on several types of cancer. A study investigated combining these factors on MDA‐MB‐231 breast cancer cells. The results showed that the combination of GA and Cur significantly reduced MDA‐MB‐231 cell growth. Furthermore, in MDA‐MB‐231 cells, this compound elevated cytotoxic activity, ROS level, and glutathione depletion. Flow cytometry analysis indicated that the compound of GA and Cur elevated the sub‐G1 cell population. Moreover, fluorescent staining and Annexin V/PI assay showed that apoptotic cells increased notably in the existence of GA and Cur. Finally, in MDA‐MB‐231 cells, protein expression found that the combination of GA and Cur notably decreased Bcl‐2 levels but increased PARP, Bax, and cleaved‐caspase 3 levels (Moghtaderi et al., 2018).

Pirarubicin (Pira) is an antitumor drug used to treat leukemia. The studies showed that GA/Pira compounds in K562 and K562/Dox cancer cells decreased cell survival, ATP levels, and mitochondrial activity in a GA concentration‐dependent manner. In GA‐treated K562/Dox cancer cells, GA suppressed P‐glycoprotein‐mediated efflux of Pira. Also, GA increased the antitumor effect of Pira on K562 and K562/Dox cancer cells by disrupting cellular energy status and reversing drug resistance in live K562/Dox cancer cells by blocking P‐glycoprotein function (Aye et al., 2021).

#### 4.1.5 Anticancer activity of GA in nanoparticle formulations

The bioactive copper‐GA nanoscale metal‐organic framework was synthesized and studied for the codelivery of GA as an anticancer factor and methylene blue as a photosensitizer for cancer cells. Synth copper‐bioactive frameworks (bio‐MOFs) were employed as carriers of two antitumor factors. GA was considered part of the framework structure (building block). Methylene blue (MB) is loaded as a guest molecule in the context of the amphiphilic pores. In vitro and in vivo assays have shown increased cytotoxicity of two nano pharmaceutical frameworks compared to equivalent doses of free drugs in the presence of light (Sharma et al., 2019).

One study redesigned the antitumor nanocomposite formulation using polyethylene glycol‐coated iron oxide nanoparticles and GA as the anticancer drug (Fe_3_O_4_‐PEG‐GA). In vitro, research indicated that characteristics and drug loading percentage were better than the previously reported formulation. The anticancer effect of GA without a nanocarrier (Fe_3_O_4_‐PEG) and compare with Fe_3_O_4_‐PEG‐GA in human breast cancer cells (MCF‐7), human lung cancer cells (A549), and human colon cancer cells (HT‐29) after incubation for 24, 48, and 72 h using MTT assay. The formulated (Fe_3_O_4_‐PEG‐GA) showed an improved anticancer effect compared to GA alone (Rosman et al., 2018).

The antitumor effect of encapsulated polyherbal nanoparticles (GA and quercetin nanocomposite) and polyherbal extract (amla and pomegranate fruit peels) were investigated in DMH‐induced colorectal cancer in rats. In normal and DMH‐induced rats, a pharmacokinetic study showed that polyunsaturated nanoparticles had a typical sustained release profile (Sustained release profile form is defined as well characterized and reproducible profile form, which is designed to control drug release profile at a specified rate to achieve desired drug concentration either in blood plasma or at the target site) with a fourfold higher bioavailability than polyunsaturated extracts. After oral application, pharmacokinetic parameters for multi‐plant nanoparticles and multi‐plant extracts were determined using a single‐compartment approach based on the serum concentrations profile of multi‐plant nanoparticles and multi‐plant extracts. This study showed that encapsulation of GA and quercetin in polymer nanoparticles enhance oral bioavailability and anticolon cancer activity (Patil & Killedar, 2021a).

One study synthesized environmentally, and low‐cost silver nanoparticles (AgNPs) using GA in bentonite/starch biocomposites (BNCs) for oral use and evaluated its antibacterial and anticancer effects. The composition of AgNPs was confirmed by the UV‐vis absorption peak at 412 nm. The results showed that AgNPs‐GA synthesized in BNCs could be a potential candidate for suppressing the growth of bacteria, and they have shown significant cytotoxicity against MCF‐7 cancer cells, too (Thapliyal & Chandra, 2018). Another study used single‐dispersed high‐performance (Ag‐Se) single‐particle nanoparticles by quercetin and GA. Bimetallic nanoparticles were synthesized at room temperature. Various reaction factors such as quercetin GA and Ag/Se salt concentration, temperature, pH, and reaction time are optimized for controlling nanoparticle properties. Different analytical techniques characterized the nanoparticles, and their size was 30–35 nm. Results suggest that flavonoids and phenolics caused nanoparticle reduction and stabilization. This research explained the efficacy of quercetin and GA‐mediated synthesis of bimetallic (Ag–Se) nanoparticles, which are antioxidant, anticancer, and antimicrobial in vitro. Synthesized Ag‐Se nanoparticles were used as antitumor factors for Dalton lymphoma cells, and results show that 80% of their viability was decreased (Mittal et al., 2014).

A study on the synergistic impact of GA from amla fruit and quercetin from pomegranate peel in chitosan for targeted delivery to colorectal cancer showed significant changes in crypt misplaced foci in CS nanoparticles compared to polyunsaturated extracts, with a significant reduction in levels of catalase, glutathione, and colonic superoxide dismutase (Patil & Killedar, 2021b).

The anticancer effects of GA alone, in combination with other drugs, and in the form of nanoformulations are summarized in Table 2.

## 5 CONCLUSION AND PERSPECTIVE

Given that in recent years, anticancer and antibacterial therapies have faced widespread drug resistance that has led to treatment failure, the use of new drugs and their combinations with low toxicity and side effects is essential (Patel et al., 2021). Currently, the use of plant compounds as antibacterial and anticancer compounds has been popular (Bhandari et al., 2017; Khameneh et al., 2019; Shrihastini et al., 2021). In this regard, phenolic compounds such as GA have shown favorable results. Hence, in this study, we described the chemical and biological properties of GA and its derivatives, their pharmacokinetics and bioavailability, and the importance of using GA and its mechanism as an antibacterial and anticancer agent. As mentioned earlier, GA is one of the most important natural phenolic compounds found in many dietary substances including various plants, fruits, and vegetables. It has antioxidant and antiinflammatory properties and has pharmacological effects on various diseases. After administration, it is absorbed by the gastrointestinal tract and after being metabolized in the liver to various compounds, is excreted by the kidneys. The toxic effects of GA have rarely been seen in animal studies and clinical trials and its safety has been confirmed, but due to the rapid absorption and metabolism of GA, its bioavailability is low which can limit its therapeutic applications. To overcome this problem, the use of biopolymers or nanoformulations to increase their stability and absorption has been proposed as a suitable method. In addition, the combination of GA with many antibacterial or anticancer drugs showed synergistic effects against multidrug‐resistant pathogens or cancer cells and improved the efficacies of both antibacterial and anticancer drugs, which can be an effective way to reduce and prevent drug resistance. Because the combination of common antibacterial and anticancer drugs with GA can reduce their effective dosage and increase the sensitivity of cells to the existing drugs (Buchmann et al., 2022). In addition, GA and its derivation alone or in combination with other drugs can reverse drug resistance in bacteria and cancer cells by inhibiting efflux pumps such as ABC transporters, MrsA, NorA, TetK pumps, and P‐glycoproteins (Dashtbani‐Roozbehani & Brown, 2021; Pereira Rangel et al., 2010). Inhibition of pumps leads to an increase in the accumulation of drugs in the resistant cells and enhances their cytotoxicity. According to what was mentioned; GA and its derivatives have potent antibacterial and anticancer action. They inhibit the growth of plankton in gram‐positive and gram‐negative bacteria by degradation of the cell morphology, increasing membrane permeability, the release of intracellular components, and enhancing the production of ROS. Moreover, they inhibit biofilm formation by inhibiting the synthesis of proteins and polysaccharides in biofilm structure.

In different cancer cells, GA induces cell death and inhibits cell growth and migration by different mechanisms, including targeting intrinsic and extrinsic signaling pathways related to apoptosis, DNA damage through DNA fragmentation, and recruit's DNA repair genes, effect on the expression of apoptotic and nonapoptotic proteins, ROS production, activation of NF‐κB, depletion of GSH, down‐regulation of MMP expression, and cell cycle targeting. Therefore, according to studies, GA as a plant phenolic compound has the potential to treat and/ or manage bacterial infections and various cancers. Hence, GA and its derivatives have been considered by researchers in various fields of drug development worldwide. Further research in this area may lead to more insights, and identification of newer functions and effects of these compounds. Given that extensive clinical studies have not been performed so far and the exact mechanism of action is still unknown, further studies in humans are necessary to confirm the results obtained at the level of in vitro and place GA as a potential drug in the commercial market.

## AUTHOR CONTRIBUTIONS

**Saeedeh Keyvani‐Ghamsari:** Conceptualization (equal); investigation (equal); methodology (equal); writing – original draft (equal); writing – review and editing (equal). **Maryam Rahimi:** Investigation (equal); methodology (equal); writing – original draft (equal). **Khatereh Khorsandi:** Conceptualization (equal); data curation (equal); methodology (equal); resources (equal); software (equal); supervision (equal); visualization (equal); writing – review and editing (equal).

## CONFLICT OF INTEREST STATEMENT

The authors declare no conflict of interest.

## Data Availability

The data presented in this study are available in this manuscript.
